# Improved Methods for the Rapid Formation and Prevention of Advanced Glycation End Products (AGEs) In Vitro by Coupling to the Hypoxanthine/Xanthine Oxidase Assay System

**DOI:** 10.3390/biomedicines6030088

**Published:** 2018-08-15

**Authors:** Samuel Marques, Teresa Trevisan, Carlos Maia, Andrea Breuer, Robert W. Owen

**Affiliations:** 1 Instituto Federal de Educação, Ciência e Tecnologia do Ceará, Departamento de Química, Av. José de Freitas Queiroz, 5000, Quixadá–CE CEP 63902-580, Brazil; samuel.marques@ifce.edu.br; 2 Campus do Pici–Bloco 935 superior–Laboratório de Produtos Naturais e 10 Biotecnologia e (LPNBio), Universidade Federal do Ceará, Fortaleza CEP 60451-970, Brazil; mariattre@hotmail.com; 3 Instituto Federal de Educação, Ciência e Tecnologia do Ceará, Departamento de Química, rua Luis Cunha, S/N–Monte Castelo, Ubajara–CE CEP 62350-000, Brazil; educadu20@hotmail.com.br; 4 National Center for Tumor Diseases (NCT)/German Cancer Research Center (DKFZ), Division of Preventive Oncology, Im Neuenheimer Feld 460, 69120 Heidelberg, Germany; andrea.breuer@nct-heidelberg.de

**Keywords:** advanced glycation end products, glyoxal, hypoxanthine/xanthine oxidase, methyl glyoxal, RCS-trapping, rutin, xanthine oxidase

## Abstract

Advanced glycation end products (AGEs) represent a set of molecules that contribute directly to the initiation and aggravation of diseases associated with ageing. AGEs are produced by the reaction between reducing sugars (or α-dicarbonyl compounds), proteins, and amino acid residues. Previous in vitro methods using non-enzymatic procedures described in the literature require an incubation period of 1–3 weeks to generate AGEs. In this study, the reaction time for the formation of AGEs (48 and 3 h) was significantly reduced by adaptation of methods previously described in the literature and coupling them to the free radical generation system termed hypoxanthine/xanthine oxidase assay. The incorporation of this assay into the experimental system accelerated the production of AGEs as a result of the formation of reactive oxygen species (ROS), as shown by increased fluorescence. The capacity of different classes of chemical compounds (aminoguanidine, chlorogenic acid, rutin, and methanol extracts of *Hancornia speciosa* Gomes) to inhibit protein glycation by acting as scavenging agents of α-dicarbonyl species was evaluated. Aminoguanidine and, especially, rutin identified in the leaf extracts of *H. speciosa* Gomes showed a high capacity to act as scavengers of reactive carbonyl species RCS-trapping, resulting in the inhibition of AGEs formation.

## 1. Introduction

The world population is undergoing a period of significant change in terms of increased life expectancy and the provision of therapeutic options for diseases that predominantly affect older people. In the field of chemistry, the evidence and elucidation of oxidative stress caused by the action of reactive oxygen species (ROS) in organisms, are significantly contributing to the understanding of several pathologies associated with the ageing process. Understanding the effects of ROS in humans has stimulated, for example, an increase in the consumption of foods with antioxidant capacities, such as fruits and vegetables [1].

Nevertheless, recent studies indicate that, in addition to ROS, another group of substances called advanced glycation end products (AGEs) contributes directly to initiate and aggravate ageing-related pathologies. Research has revealed a crucial relationship between the formation of AGEs (glycation of proteins) and various age-related diseases such as diabetes, atherosclerosis, renal disorders, and neurodegenerative diseases such as Alzheimer’s disease [2,3]. In vivo, AGEs cause non-enzymatic modifications of proteins’ structure, affecting their functions in numerous ways and causing the aforementioned diseases.

The endogenous and exogenous production of AGEs is explained by the reaction mechanism that gives aroma and color to processed foods, also known as the Maillard reaction. It consists of a complex series of parallel and subsequent chemical reactions (oxidation and condensation) that produce an wide class of compounds described by the French biochemist Louis Camille Maillard [4,5,6,7,8]. In this process, the formation of crosslinks between the amino acid residues of proteins and carbonyl species, that are produced by human metabolism or through the processing of food, gives rise to different chemical adducts (AGEs).

**Figure 1:**
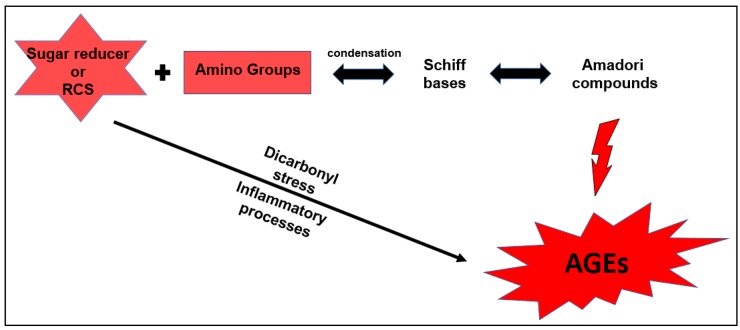
Schematic simplification for the formation of advanced glycation end products (AGEs). RCS: reactive carbonyl species.

Initially, a condensation reaction occurs, in which the carbonyl groups of reducing sugars (or α-dicarbonyls formed by their fragmentation) undergo nucleophilic attack on the non-ligand electron pair of amino groups present in proteins or amino acid residues (arginine, lysine, etc.) [4,5]. These condensation reactions produce intermediate carbinolamines called Schiff bases. Then, through a metameric effect and oxidation and dehydration reactions, the Schiff bases become more stable species, termed Amadori compounds. Finally, the Amadori compounds produce AGEs (Figure 1) [4,5,6,7,8,9,10].

The Amadori products can give rise to new protein residues through oxidative fission or retro-aldol fragmentation, which react with α-dicarbonyl compounds and thus increase the amount of AGEs. Depending upon the pH of the environment, the Amadori compounds may also undergo enolization and produce other α-dicarbonyl compounds. During inflammatory processes, excessive formation of dicarbonyl compounds (RCS) occurs through the action of the enzymes myeloperoxidase and NADPH oxidase, promoting a biochemical state known as dicarbonylic stress [11]. The presence, in excess, of these dicarbonyl species favors further formation of AGEs in humans [4,5,6,7,8,9,10,11,12,13].

The reaction between dicarbonyl compounds, such as methylglyoxal (MGO) and glyoxal (GO), with proteins or amino acid residues promotes the glycation process more rapidly than the reaction of the sugars in their open form. When in excess in the human body (mainly during inflammatory processes), these dicarbonyl species generate a biochemical process known as``dicarbonyl stress” that induces harmful effects in humans due to an increase in AGEs formation [4,5].

Chemically, AGEs are covalently linked (fluorescent and non-fluorescent) protein adducts or cross-linking products between different amino acid residues. Several techniques can be used for the identification and quantification of AGEs, such as, in particular, spectrofluorimetry, liquid and gas chromatography coupled to mass spectrometry, and ELISA(enzyme-linked immunosorbent assay) [5,13].

Numerous studies have been carried out in the last two decades to produce AGEs in vitro in order to elucidate their main pathways of formation, chemical structures, and biologic effects [13]. The procedures currently described in the literature to obtain AGEs, using non-enzymatic reaction pathways, require one to three weeks for their formation [14,15].

Several authors have proposed methods to inhibit the formation of AGEs, analyzing the capability of various natural and synthetic chemical compounds to act as scavengers of α-dicarbonyl species (RCS-Trapping) and thus minimize dicarbonyl stress. Guanidines (aminoguanidine), catechins (epigallocatechin), proanthocyanidins, and curcumin are among some of the classes of substances capable of inhibiting the formation of AGEs by acting as α-dicarbonyl species scavengers (RCS-Trapping) [16,17,18,19,20,21].

It is known that the production of AGEs, in vivo, is linked to the generation of ROS that promote the glycation process of proteins. Despite this evidence, only a very small number of studies relating the generation of ROS to the formation of AGEs have been carried out to date, even though both ROS and AGEs are related to oxidative stress and consequently to premature ageing [22,23,24,25]. In this context, the coupling of the procedures for the formation of AGEs (in vitro) to an environment with high ROS generation can promote a significant reduction in the time required to evaluate the antiglycation capacity of chemical compounds and accelerate the search for proactive drugs.

Therefore, a major aim of this study was to reduce the reaction time for AGEs formation by adaptation of methods already reported in the literature [26,27,28,29,30,31] and coupling them to a ROS-generating system termed the hypoxanthine/xanthine oxidase assay [28,30]. In this method, hydroxyl radicals are produced by the oxidation process of hypoxanthine to uric acid by the action of the enzyme xanthine oxidase (XO) on hypoxanthine, as described by Owen and collaborators [29,31]. The presence of ROS in excess during the glycation and glycooxidation of the protein can promote the oxidation of amino acid residues as well as sugars, leading to the rapid formation of dicarbonyl derivatives and significantly reducing the time of the glycation process of proteins.

The final procedures developed to produce AGEs using the reaction pathways with sugars (glucose and fructose) or dicarbonyl compounds (MGO and GO) are termed hypoxanthine/XO–bovine serum albumin (BSA)–glucose/fructose and hypoxanthine/XO–BSA–MGO/GO assay systems, respectively.

Different classes of chemical compounds (aminoguanidine, chlorogenic acid, and rutin), as well as methanol extracts of leaves, bark, and fruits of *Hancornia speciosa* Gomes (HS) were evaluated for their capacity to inhibit the glycation process.

## 2. Experimental Section

### 2.1. Reagents

Hydrochloric acid, sodium chloride, bovine serum albumin (BSA), glucose, fructose, methylglyoxal, glyoxal, Trizma, salicylic acid, cinamic acid, aminoguanidine hydrochloride, chlorogenic acid, and rutin were obtained from Sigma-Aldrich Chemie (Steinheim, Germany); acetonitrile was from Fluka/Riedel de Haen (Seelze, Germany); acetic acid, dimethyl sulfoxide (DMSO), EDTA, ferric chloride hexahydrate, hypoxanthine, methanol, xanthine, and xanthine oxidase (XO) were from Merck (Darmstadt, Germany); K_2_HPO_4_ and KH_2_PO_4_ were from Serva (Heidelberg, Germany). All solutions were made in double distilled water, DMSO, or methanol.

### 2.2. General Description of the Methods Developed for the Formation and Inhibition of Advanced Glycation Products (AGEs)

Maillard’s description demonstrates that the reaction between reducing sugars in their open form (or of carbonyl compounds generated by their oxidation) with proteins or amino acid residues produces Amadori compounds, which are converted into AGEs. Therefore, the methods presented in this study consisted in initially studying the reaction of sugar solutions (glucose and fructose) with a BSA solution in different reaction media and subsequently replacing the sugar solutions with dicarbonyl compounds, namely, glyoxal (GO) and methylglyoxal (MGO), with the addition of xanthine oxidase (XO) to promote the formation of AGEs in a reaction medium enriched with hydroxyl free radicals (HO^●^).

Enzyme-catalyzed reactions have rate constants that are 100,000 times higher than non-enzymatic reactions and are therefore physiologically meaningful. The qualitative formation of AGEs was verified by spectrofluorimetric measurements, with excitation and emission at wavelengths of 350 and 450 nm, respectively [5,13].

The final procedures developed to produce AGEs using the reaction pathways with sugars (glucose and fructose) or dicarbonyl compounds (MGO and GO) were termed hypoxanthine/XO–BSA–glucose/fructose and hypoxanthine/XO–BSA–MGO/GO assay systems, respectively.

### 2.3. Formation of AGEs Induced by the Hypoxanthine/XO­–BSA–Glucose/Fructose System

Initial exploratory experiments to evaluate the formation of AGEs in vitro were conducted with various reaction buffers (see Supplementary Material).

The final buffer solution selected comprised 500 mL of phosphate buffer (KH_2_PO_4_ + K_2_HPO_4_, 0.1 M, pH = 6.6), EDTA (73 mg), FeCl_3_6H_2_O (33 mg), and hypoxanthine (20.5 mg). The reaction mixtures added to Eppendorf microfuge tubes (2.0 mL) contained 94 µL of BSA (16 mg/mL buffer), 25 µL of glucose (1.67 M in buffer), and 882 µL of phosphate buffer. The incubation was conducted in the absence of light at 37 °C with constant stirring (450 rpm) for 24 h, 48 h, 1 week, 2 weeks, and 3 weeks) [26,27,28,29,30]. After the incubation reactions were terminated by the addition of 10 µL of concentrated hydrochloric acid, aliquots were transferred to 96-well plates, and measurements of AGEs total fluorescence intensity were performed by reading the microplates in a Cytoflour fluorescence spectrophotometer (Perseptive Biosystems Inc., 500 Old Connecticut Path, Framingham, MA, USA) with excitation and maximum emission at 350 and 450 nm, respectively. The analyses were conducted in duplicate, and the fluorescence measurements were performed in triplicate.

After verification of the formation of AGEs in this system, the same procedure described above was repeated with the addition of 10 µL of xanthine oxidase (XO; 18 mU-Merck, Darmstadt, Germany) to the reaction mixtures. In all assays, controls were set up in the absence of XO for comparison.

To evaluate the reactivity of different sugars, glucose was replaced by the same amount of fructose (1.67 M). Initially, the incubation period was 48 h (37 °C), in the presence or absence of XO. This procedure was repeated for different incubation periods and temperatures: one week (37 °C) and 48 h (90 °C), in the absence of XO.

### 2.4. Formation of AGEs Induced by the System Hypoxanthine/XO–BSA–MGO/GO

This method was basically the same as that described for glucose and fructose (sugar + BSA + buffer ± enzyme), but the sugar solutions were replaced by equal amounts (25 µL) of GO and MGO (5.55 × 10^−2^ and 6.89 × 10^−2^ M, respectively) dissolved in 0.1 M phosphate buffer (pH = 6.6).

To Eppendorf microfuge tubes (2.0 mL), 94 µL of BSA (16 mg/mL), 25 µL of MGO or GO solutions (5.55 × 10^−2^ and 6.89 × 10^−2^ M, respectively), and 882 µL of phosphate buffer (pH = 6, 6) were added. The reactions were conducted in duplicate and analyzed in the presence or absence of 10 µL XO (18 mU), with incubation at 37 °C and 450 rpm for 3 h. Controls were produced by replacing the BSA with an equal amount of phosphate buffer (pH = 6.6). The reaction completion procedure (addition of 10 µL of concentrated HCl) and determination of fluorescence were conducted in the same manner as for the hypoxanthine/XO–BSA–glucose/fructose assay.

### 2.5. Inhibition of AGEs Formation

The procedures with glucose, MGO, and GO were repeated with the purpose of analyzing the capacity of different classes of compounds (guanidines, flavonoids, and phenolic acids) and plant extracts to inhibit the formation of AGEs. For this, different pure compounds (aminoguanidine, rutin, and chlorogenic acid) at varying concentrations as well as methanol extracts of the leaves, bark, and fruits of HS were added to the reaction mixtures. The aim of this was to demonstrate the possible action of these chemical species as scavengers of the 1-2-dicarbonyl compounds MGO and GO, acting as trapping agents (RCS-trapping) of these species to form adducts and leading to a decrease in the amount of AGEs [16,32,33,34,35,36,37,38,39,40].

As a positive control for the methods developed to inhibit the formation of AGEs, aminoguanidine hydrochloride (CH_6_N_4_·HCl) at 98% purity (Sigma Aldrich) was used. Methanol solutions of the compounds were prepared at different concentrations (0.5–5.0 mM). Subsequently, 1.0 mL of these solutions were added to Eppendorf microfuge tubes (2.0 mL), and the methanol was removed on a Speedvac (M + S Laborgerate GMBH, 69168 Wiesloch, Germany). After removal of the solvent, the residues were dissolved in the appropriate buffers and incubated at 37 °C for 48 h (sugar buffer) and 3 h (MGO/GO buffer).

Methanol extracts of HS leaves, bark, and fruits were also evaluated. Solutions of the extracts (10 mg/mL) were prepared, 0.5 mL (5 mg of extract) was added to Eppendorf microfuge tubes (2.0 mL), and the solvent was removed on a Speedvac ((M + S Laborgerate GMBH, 69168 Wiesloch, Germany). The residues were dissolved in the sugar buffer and incubated at 37 °C for 48 h in the presence or absence of XO.

Only the results obtained for the leaf methanol extract of HS showed a reduction in fluorescence intensity when compared to the control, which was subjected to more detailed analysis. The procedure was the same as for the pure compounds, except for using different amounts of the extract (2.5–20 mg).

Hexane, 5%, 10%, 25%, and 50% MeOH in acetic acid (2%) fractions of the methanol extract of the HS leaves obtained by fractionation on SPE C18 columns (Octadecyl-modified silica, Chromabond) were also evaluated. This test was performed with glucose, using 5.0 mg of the fractions, only in the presence of XO. Finally, the same procedures were repeated with rutin and chlorogenic acid, the major compounds detected in HS leaf extracts, in the concentration range of 250–2000 μM. In all procedures performed (pure compounds or extracts), the samples were centrifuged at 13,000 rpm for 5 min prior to fluorescence determination.

## 3. Results

The fluorescence intensities presented in all Figures of this work refer to the mean of duplicate samples, measured in triplicate. It should be noted that there was no significant discrepancy between the data obtained.

### 3.1. Formation of AGEs Induced by the System Hypoxanthine/XO–BSA–Glucose/Fructose

**Figure 2:**
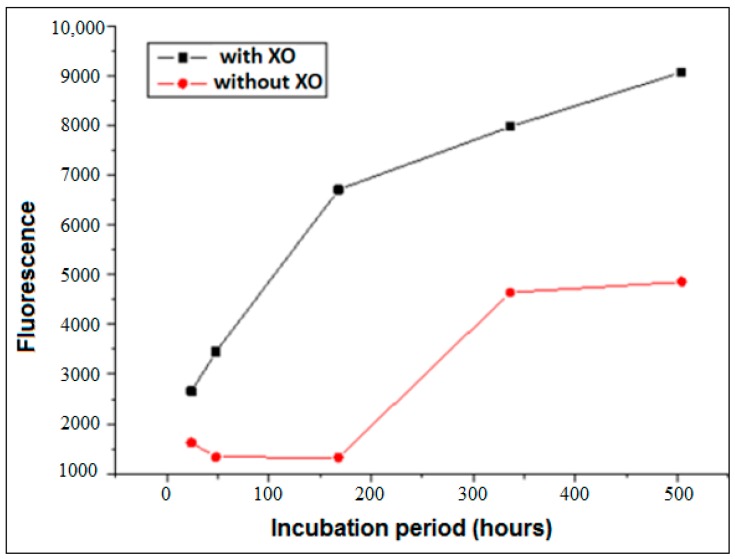
Formation of AGEs using glucose (37 °C) in the presence or absence of xanthine oxidase (XO) in pure phosphate buffer (pH = 6.6) during different incubation times (24 h, 48 h, 1 week, 2 weeks, 3 weeks).

The analysis of the reactions occurring in the presence or absence of XO for different times (24, 48 h, 1 week, 2 weeks, and 3 weeks) using glucose, is shown in Figure 2. Here, it is evident that the generation of free radicals accelerates the formation of AGEs, considering that for all the reaction times evaluated, almost a doubling or more of fluorescence was observed in the samples in which XO was added. For example, the analysis of the procedure after a one-week (168 h) incubation period revealed an almost 6-fold increase in fluorescence after the addition of XO.

**Figure 3:**
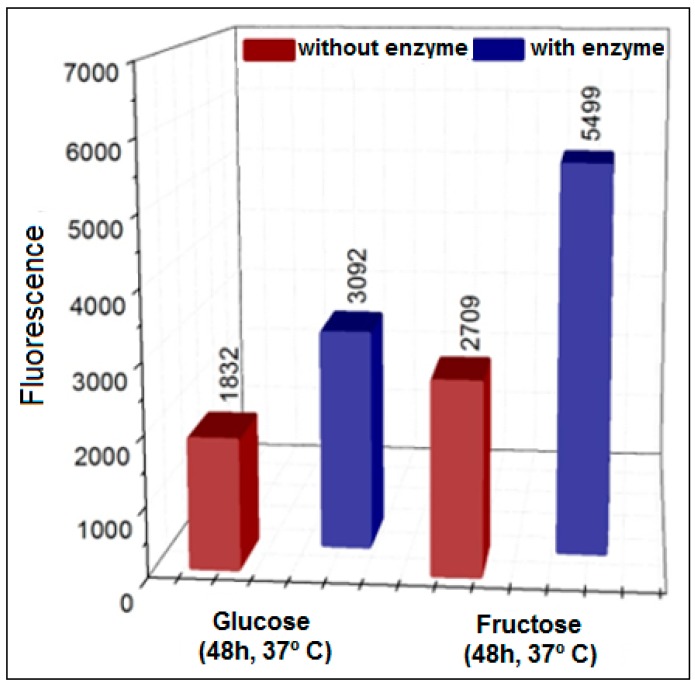
Formation of AGEs using glucose and fructose in the presence or absence of XO in pure phosphate buffer (pH = 6.6) with 48 h incubation at 37 °C.

**Figure 4:**
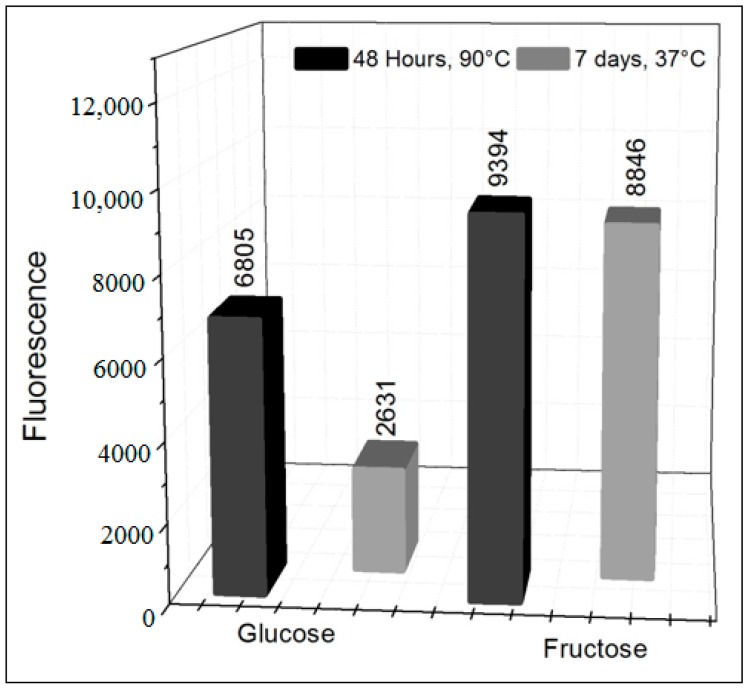
Formation of AGEs using glucose and fructose in the absence of XO in pure phosphate buffer (pH = 6.6) with incubation for 48 h (90 °C) and 1 week (37 °C).

In experiments where glucose was replaced by fructose (Figure 3), there was a considerable increase in the formation of AGEs compared to the glucose method. This result was repeated under different reaction conditions (Figure 4), indicating that fructose (ketose) is more reactive than glucose (aldose) for the formation of AGEs, as already reported [9,27,41].

On the basis of the theoretical studies of organic chemistry, the carbonyl groups of aldehydes are more reactive than those of ketones if we consider a condensation reaction that produces Schiff bases with subsequent formation of AGEs (as described for the Maillard reaction). The more pronounced reactivity of the aldoses can be justified by the more electrophilic character of the aldehyde carbonyl carbon, since it has a number of inductive electron-receiving effects smaller than those of a ketone. In addition, the carbonyl of an aldehyde has a smaller space impediment around itself, facilitating its reaction with a nucleophile [9]. However, what happens in experimental procedures and in human metabolism (in vivo and in vitro), is exactly the opposite, that is, the formation of AGEs increases significantly when glucose (aldose) is substituted by fructose (ketose), and this is confirmed by the results of this work [5,13,42].

The data obtained indicate the critical and harmful effects that fructose may have on human health. Fructose is a main carbohydrate in the human diet, especially because of the addition of fructose syrup to industrialized beverages [4,13]. This situation becomes more critical in people with hyperglycemia, such as those suffering from diabetes mellitus, in whom the blood sugar levels are naturally elevated, leading to increased formation of AGEs.

Figure 4 also indicates that the food cooking process may also contribute significantly to the formation of AGEs, since all the experiments performed at 90 °C (with glucose and fructose) showed almost double fluorescence values when compared to the experiments performed at 37 °C. This indicates that the cooking process may significantly increase the amount of AGEs in food, which corroborates some theories linked to vegetarianism that advocate the consumption of raw foods as a way of preserving health. The support of raw food consumption is based on the presence of antioxidant species in these foods as well as on the absence of AGEs formation from the cooking process.

### 3.2. Formation of AGEs Induced by the Hypoxanthine/XO–BSA–MGO/GO System

**Figure 5:**
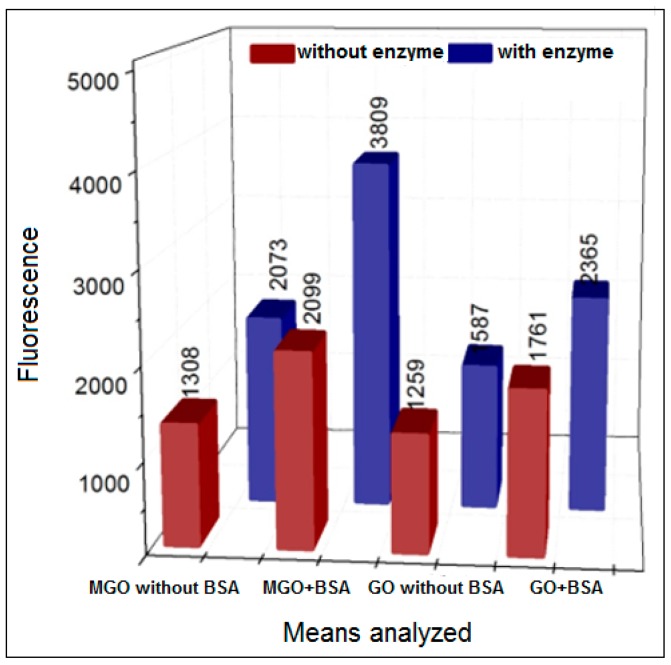
Formation of AGEs using methylglyoxal (MGO) and glyoxal (GO) (37 °C) in the presence or absence of XO in pure phosphate buffer (pH = 6.6) with 5 h incubation.

In Figure 5, it can be seen that, in comparison to the controls with intrinsic fluorescence from both MGO and GO, the increase of fluorescence in the presence of BSA due to AGEs formation is comparable to that observed with our standard sugar procedure. However, this was the case only after 3 h of incubation in comparison to the 48 h incubation of the sugar procedure.

**Figure 6:**
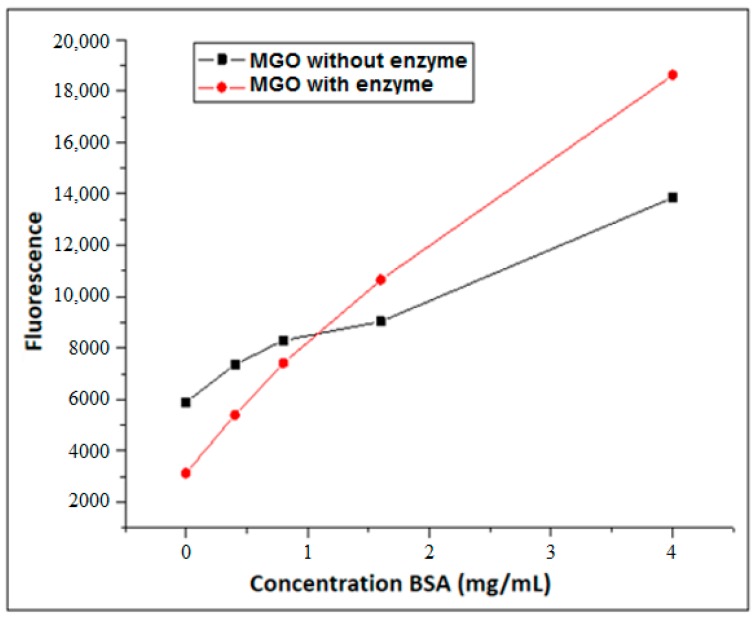
Formation of AGEs using MGO (37 °C) in the presence or absence of XO in pure phosphate buffer (pH = 6.6) with 1 h incubation and varying concentrations of BSA (0–4 mg/mL).

When MGO was incubated in the presence of BSA in the concentration range of 0–4 mg/mL at 37 °C for one hour with or without XO, a superior linearity was evident in the presence of XO, showing the importance of the controlled generation of ROS (Figure 6).

### 3.3. Inhibition of AGEs Using Pure Compounds and Methanol Extracts Plus SPE Fractions of HS

#### 3.3.1. Inhibition Using Aminoguanidine (Positive Control)

As demonstrated by Nilsson [35] and Thornalley [36], aminoguanidine (AG) may act as a positive control in AGEs assays. Aminoguanidine reacts rapidly with dicarbonyl compounds (such as MGO and GO) acting as a scavenging agent.

**Figure 7:**
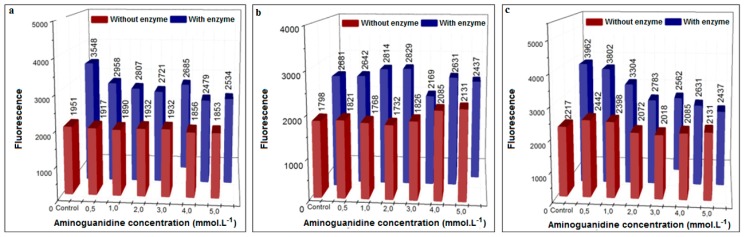
Inhibition of AGEs formation using different concentrations of aminoguanidine (37 °C) in the presence or absence of XO in pure phosphate buffer (pH = 6.6). (**a**) Procedure with glucose, 48 h incubation; (**b**) procedure with MGO, 3 h incubation; (**c**) procedure with GO, 3 h incubation.

Aminoguanidine (AG) worked moderately well (Figure 7) in the presence of XO but was ineffective in its absence and, at a concentration of 5.0 mM, was more effective in the GO assay (38% inhibition) than in the glucose (29% inhibition) and MGO (9% inhibition) assays. This compares well with the data of Thornalley (2003) [36], where 25.0 mM aminoguanidine was required to demonstrate inhibition.

#### 3.3.2. Inhibition Using HS Extracts and SPE Fractions

**Figure 8:**
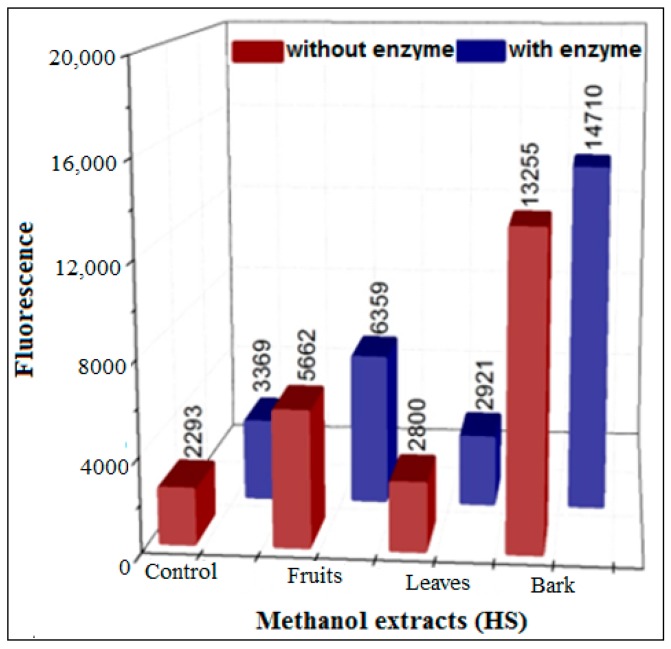
Inhibition of AGEs formation using 5 mg/mL of different HS methanol extracts with glucose (37 °C), in the presence or absence of XO in pure phosphate buffer (pH = 6.6) with incubation for 48 h.

Literature surveys revealed that leaf extracts of HS are used in traditional medicine to treat diabetes and contains rutin as a major phytochemical in organic extracts. Therefore, the capacity of methanol extracts of the leaves, bark, and fruit of HS to inhibit AGEs formation was evaluated in the glucose assay (Figure 8). Whereas the bark and fruit extracts showed significant intrinsic fluorescence, the leaf extract was characterized by low intrinsic fluorescence and was effective in the presence of XO. Therefore, this extract was studied in more depth using the three newly developed methods, only in the presence of XO.

**Figure 9:**
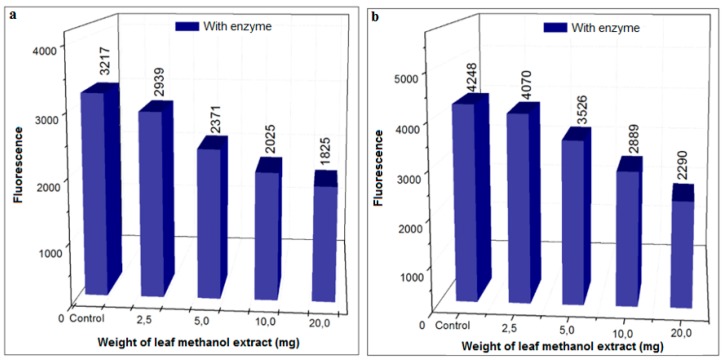
Inhibition of AGEs formation using different concentrations of methanol leaf extract of HS (37 °C) in the presence of XO in pure phosphate buffer (pH = 6.6). (**a**) Procedure with glucose, 48 h incubation; (**b**) procedure with MGO, 3 h incubation.

A dose (2.5–20.0 mg/mL of HS leaf extract)-dependent decrease in AGEs formation was observed in both the MGO and sugar assays (Figure 9). At 20 mg/mL, inhibition was similar at 43% and 46% compared to the controls, in the sugar and MGO assays, respectively.

**Figure 10:**
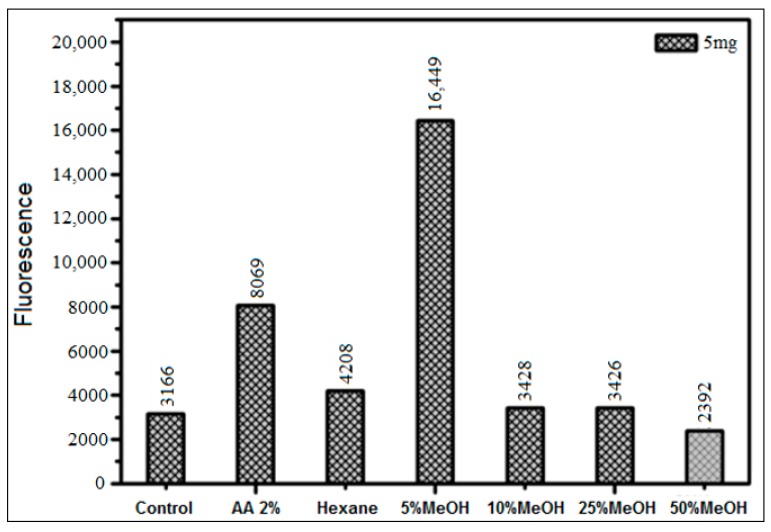
Inhibition of AGEs formation using 5 mg of various fractions obtained by Solid Phase Extraction SPE C18 columns of the methanol extract of HS leaves, measured in the glucose assay (37 °C) only in the presence of XO enzyme in pure phosphate buffer (pH = 6.6), with incubation for 48 h.

Therefore, fractions obtained by fractionation of the HS leaf extract on SPE C18 columns were also assayed. The only fraction which inhibited (24%) AGEs generation was the one eluted using 50% methanol in 2% aqueous acetic acid containing predominantly rutin (Figure 10). By contrast, the 5% methanol fraction increased AGEs generation by over five times. This fraction contained predominantly chlorogenic acid. Therefore, authentic standards of both rutin and chlorogenic acid were studied by the new assay systems. The presence of these constituents in the different fractions evaluated was evaluated by HPLC-ESI-MS by Marques (2017), who identified a range of polyphenolic compounds (*n* = 44) in the various botanical parts of HS (bark, leaves, and fruits) [8].

#### 3.3.3. Inhibition Using the Flavonoid Rutin

The ability of some flavonoids to act as scavenging agents of 1,2-dicarbonyl compounds has previously been demonstrated using procedures with an incubation period of 1–3 weeks. The compounds (+)-catechin, (−)-epicatechin, and procyanidins are reported to be active inhibitors [16,34,35]. It has also been demonstrated that (−)-epigallocatechin-3-gallate (EGCG) is a good scavenger of MGO and GO under neutral or slightly alkaline pH conditions, producing mono- and di-substituted adducts (3:1 molar ratio) from condensation reactions [38]. These reactions occur preferentially at the C6 and/or C8 positions of the A ring. The higher reactivity of these positions is attributed to the increase in nucleophilicity of these carbons due to resonance effects, activated by the phenolic groups through a condensation reaction [38].

**Figure 11:**
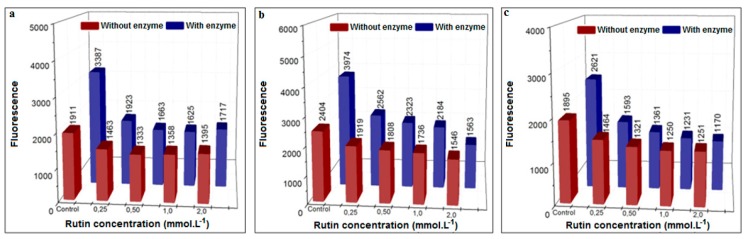
Inhibition of AGEs formation using different concentrations of rutin (37 °C) in the presence or absence of XO in pure phosphate buffer (pH = 6.6). (**a**) Procedure with glucose, 48 h incubation; (**b**) procedure with MGO, 3 h incubation; (**c**) procedure with GO, 3 h incubation.

Rutin is a flavonoid with a structure very similar to EGCG, in which gallate is replaced by a rhamnoglucoside group. In all the assays performed (glucose, MGO, and GO), rutin presented a dose–response capacity to scavenge 1,2-dicarbonyl compounds and consequently inhibit the formation of AGEs (Figure 11). As opposed to the positive control aminoguanidine, the decrease in fluorescence occurred in both the presence and the absence of XO.

Of interest is that, in the presence of XO, rutin was very effective at a concentration as low as 0.25 mM, exerting an inhibition capacity of 43%, 36%, and 39% in the glucose, MGO, and GO assays, respectively; in addition, increasing the concentration to 2.0 mM increased the inhibition by only a further 6%, 25%, and 16%. Therefore, it can be concluded that rutin is a far more effective inhibitor of AGEs formation than the positive control aminoguanidine.

#### 3.3.4. Inhibition Using Chlorogenic Acid

**Figure 12:**
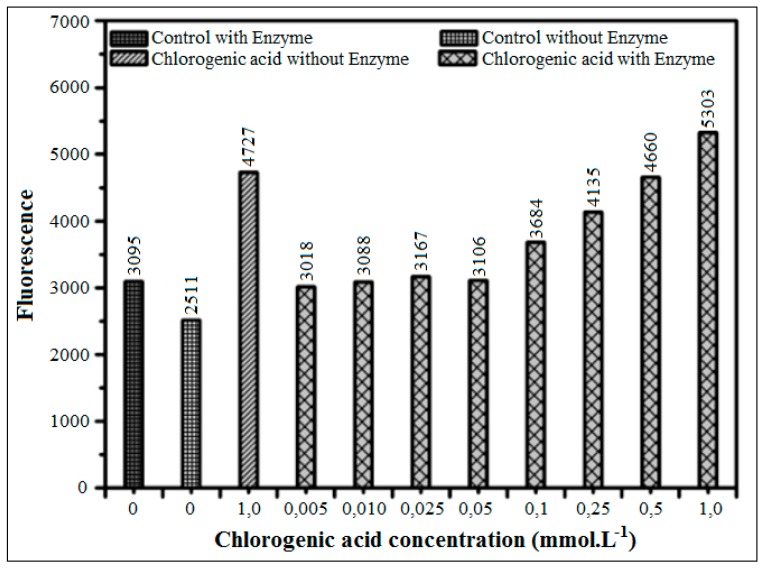
Inhibition of AGEs formation using different concentrations of chlorogenic acid determined by the glucose assay (37 °C) in the presence or absence of XO in pure phosphate buffer (pH = 6.6) with incubation for 48 h.

The inhibition capacity of chlorogenic acid, a major polyphenol detected in the 5% methanol SPE fraction, was analyzed. The data showed conclusively (Figure 12) that, in the glucose assay, chlorogenic acid could not be evaluated, due to its high intrinsic fluorescence.

The data obtained for non-fluorescent species (HS leaf extracts, aminoguanidine, rutin), indicated that the generation of AGEs in hydroxyl radical-rich reaction media, can be evaluated with incubation periods far shorter than those currently described in the literature. This will facilitate the future screening of many plant extracts and purified compounds therefrom and thus accelerate the identification of proactive antiglycation chemicals.

The problems with compounds that display high intrinsic fluorescence can be overcome by HPLC-ESI-MS techniques.

## 4. Conclusions

It is concluded that coupling of a ROS-generating system, such as the hypoxanthine/xanthine oxidase assay, to existing published methods promotes and accelerates the formation of AGEs in vitro. The assay time by the classical traditional method is reduced from 1–3 weeks to 48 h and, when glucose is replaced by MGO and GO, to only 3 h. Because this acceleration occurs in the presence of XO, it indicates very strongly that ROS are important in the generation of AGEs. It is noteworthy that, in these newly developed assays, rutin is demonstrated to be a far more efficient inhibitor of AGEs formation than the positive control aminoguanidine. 

Our data is supported by a recent clinical study showing that serum XO levels are significantly increased in type 2 diabetes mellitus patients with and without diabetic peripheral neuropathy in comparison to healthy controls. Xanthine oxidase activity was also directly correlated to the formation of AGEs in these patients [43].

## Supplementary Materials

The following are available online at http://www.mdpi.com/2227-9059/6/3/88/s1.
