# Supplementary material for: Improved Methods for the Rapid Formation and Prevention of Advanced Glycation End Products (AGEs) In Vitro by Coupling to the Hypoxanthine/Xanthine Oxidase Assay System
Source: Biomedicines. 2018 Aug 15;6(3):88. doi: 10.3390/biomedicines6030088 (PMC6164639; doi:10.3390/biomedicines6030088)
Supplement: Supplementary file 1 [file biomedicines-06-00088-s001.zip › biomedicines-339368-SI.pdf]

biomedicines

Improved Methods for the Rapid Formation and Prevention of Advanced Glycation End Products (AGEs) In Vitro by Coupling to the Hypoxanthine/Xanthine Oxidase Assay System

After the determination of the coupling efficiency of the medium containing xanthine oxidase (which promotes the generation of ROS), this procedure was repeated with 24 and 48 h of incubation, and again only with the phosphate buffer (pH = 6.6) in three distinct formulations (in the absence or presence of cinnamic acid or salicylic acid). Controls in the absence of XO were used for comparison.

Because of the formation of fluorescent compounds resulting from hydroxylation of salicylic and cinnamic acids by hydroxyl radicals, which caused interference in the measurement of the fluorescence of the AGEs, only the phosphate buffer (pH = 6.6) was used for all further experiments.

The intrinsic fluorescence of MGO and GO was initially determined using a calibration curve in the range of 0–400 mg/mL in phosphate buffer (pH = 6.6).

To evaluate the linearity of the processes in the presence and absence of XO, a similar test to the previous one was performed, in which the protein concentration (BSA, 0-4 mg/mL) was varied, maintaining the concentration of MGO at 5.55×10^{-2} M. The samples were evaluated in the presence or absence of XO during one hour of incubation at 37 °C. With this experiment, we also sought to verify the reproducibility of the method using XO. As a way of avoiding the verification of false positive results, samples not showing an increase of fluorescence when compared to the control were produced in the absence of one of the reaction components (MGO, GO, or BSA). The same procedure was performed in sugar (glucose/fructose) assays.

Four different reaction media were initially tested to establish the optimum conditions for AGEs formation in vitro, using the same concentration of glucose and BSA. The results based on the increase of fluorescence minus the control values after one-week incubation at 37 °C are shown in Figure S1a. The data also shows that the K_2HPO_4 (0.1 M) and phosphate buffer (0.1 M) (pH = 6.6) containing EDTA, $FeCl_3 6H_2O$, hypoxanthine, and salicylic acid were far superior to Tris buffer and KH_2PO_4. Therefore, phosphate buffer (0.1 M) (pH = 6.6) containing EDTA, $FeCl_3 6H_2O$, hypoxanthine, and salicylic acid was used for all further experiments.

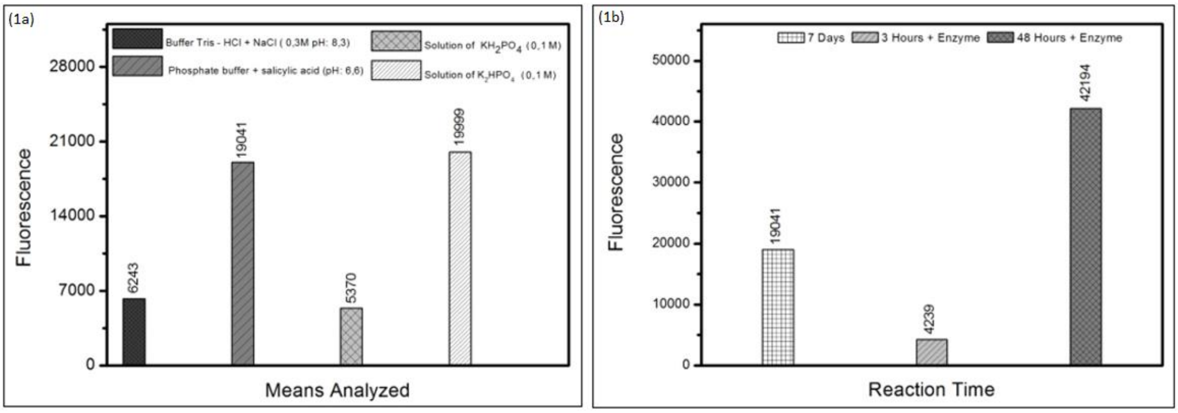

Figure S1. Formation of AGEs using glucose at 37 °C without the addition of XO (classic method) (**a**) in different media, evaluated after one week of incubation, (**b**) in phosphate buffer (pH = 6.6) with salicylic acid, with different incubation periods.

The data for experiments performed with the addition of XO to this buffer system enzyme after 3 and 48 h of incubation at 37 °C are shown in Figure S1b in comparison to 7 days incubation in the absence of XO. The efficiency of coupling the free radical generation system to generate AGEs is evident, considering that, after only 48 h of reaction (using XO), there is an increase of over 122% in the formation of AGEs in comparison to the methods currently used, which require at least one week to produce such results. It should also be noted that, in the procedure with XO, performed for only 3 h, already 25% fluorescence is produced compared to the traditional methods that require seven days.

A comparison of three different compositions of phosphate buffer pH 6.6 (1. EDTA, $FeCl_3$, hypoxanthine, salicylic acid; 2. EDTA, $FeCl_3$, hypoxanthine, cinnamic acid; 3. EDTA, $FeCl_3$, hypoxanthine) in the procedure with glucose with 24 and 48 h of incubation (Figure S2) revealed a significant increase in fluorescence in the reaction mixtures in which either salicylic or cinnamic acids were added. This elevation can be explained by the formation of fluorescent compounds from the reaction between hydroxyl radicals and salicylic and cinnamic acids (formation of 2,3- and 2,5-dihydroxybenzoic acid (DHBA) and the constitutional isomers of cinnamic acid), as demonstrated by Owen et al. [29] and confirmed by HPLC in these experiments. The major contribution to this increase in fluorescence was due to 2,5-DHBA and *o*-coumaric acid formed by hydroxyl radical attack on salicylic acid and cinammic acid, respectively, as shown in Figure S3. In order to avoid interference in the measurement of AGEs, only phosphate buffer (pH = 6.6) plus EDTA, $FeCl_3$, and hypoxanthine, in the absence of salicylic and cinnamic acids, was used for further experimentation.

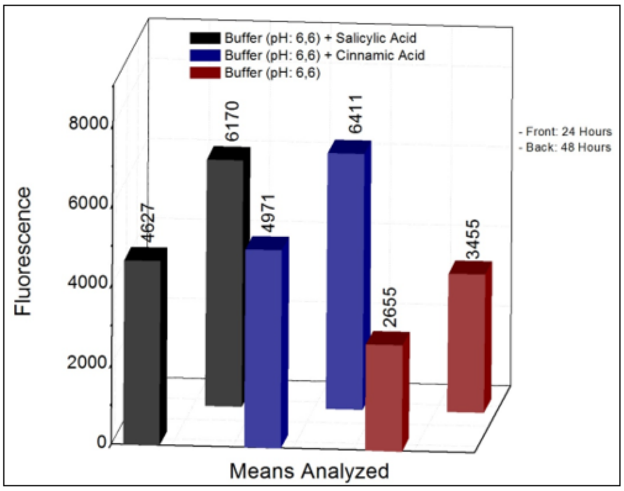

Figure S2. Formation of AGEs using glucose (37 °C) with different phosphate buffer compositions (pH = 6.6), with addition of XO and incubation at 37 °C for 24 and 48 h.

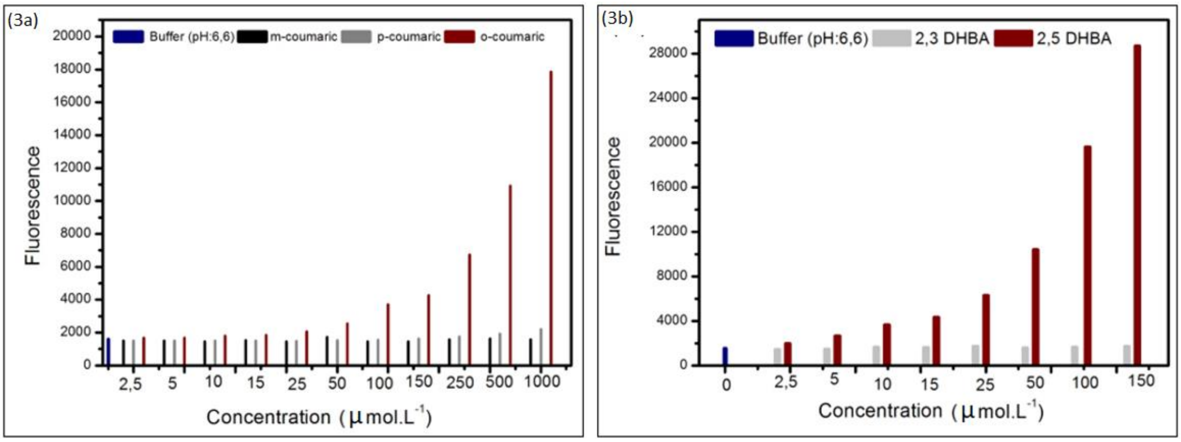

Figure S3. Intrinsic fluorescence at different concentrations of pure phosphate buffer (pH = 6.6): (**a**) constitutional isomers of coumaric acid (**b**) 2,3- and 2,5-dihydroxybenzoic acids (DHBA).

The calibration curves of MGO and GO (0–400 mg) demonstrating the intrinsic fluorescence are shown in Figure S4. Here, high fluorescence is observed for MGO, which could indicate an interference factor in the quantification of AGEs. As a way of avoiding this possible interference, controls were used in the absence of BSA, to subtract the intrinsic fluorescence of the MGO and GO contained in the reaction mixture.

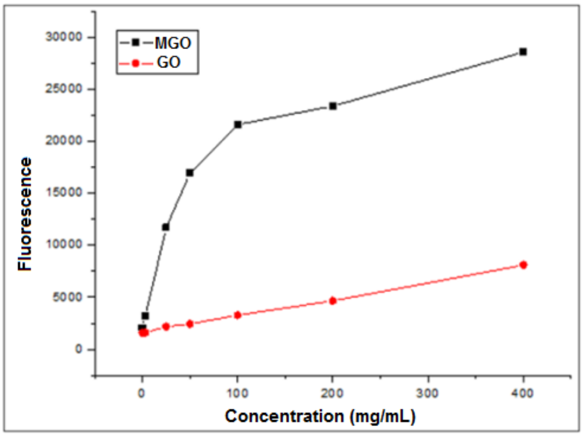

Figure S4. Intrinsic fluorescence at different concentrations of MGO and GO in pure phosphate buffer (pH = 6.6).
